# Multiple Bio-Inspired Father–Son Underwater Robot for Underwater Target Object Acquisition and Identification

**DOI:** 10.3390/mi13010025

**Published:** 2021-12-26

**Authors:** Ruochen An, Shuxiang Guo, Yuanhua Yu, Chunying Li, Tendeng Awa

**Affiliations:** 1 Graduate School of Engineering, Kagawa University, Takamatsu 761-0396, Japan; s19d501@stu.kagawa-u.ac.jp (R.A.); s21d503@kagawa-u.ac.jp (C.L.); s20g517@stu.kagawa-u.ac.jp (T.A.); 2 Key Laboratory of Convergence Medical Engineering System and Healthcare Technology, Ministry of Industry and Information Technology, School of Life Science and Technology, Beijing Institute of Technology, Beijing 100081, China; 3 School of Life Science and Technology, Changchun University of Science and Technology, Changchun 130022, China; yuyuanhua8888@126.com

**Keywords:** father–son underwater robot system, spherical underwater robot, bionic robot, hydrodynamic analysis

## Abstract

Underwater target acquisition and identification performed by manipulators having broad application prospects and value in the field of marine development. Conventional manipulators are too heavy to be used for small target objects and unsuitable for shallow sea working. In this paper, a bio-inspired Father–Son Underwater Robot System (FURS) is designed for underwater target object image acquisition and identification. Our spherical underwater robot (SUR), as the father underwater robot of the FURS, has the ability of strong dynamic balance and good maneuverability, can realize approach the target area quickly, and then cruise and surround the target object. A coiling mechanism was installed on SUR for the recycling and release of the son underwater robot. A Salamandra-inspired son underwater robot is used as the manipulator of the FURS, which is connected to the spherical underwater robot by a tether. The son underwater robot has multiple degrees of freedom and realizes both swimming and walking movement modes. The son underwater robot can move to underwater target objects. The vision system is installed to enable the FURS to acquire the image information of the target object with the aid of the camera, and also to identify the target object. Finally, verification experiments are conducted in an indoor water tank and outdoor swimming pool conditions to verify the effectiveness of the proposed in this paper.

## 1. Introduction

Underwater robots need to be commonly equipped with the manipulator for the task of executing acquisition [1,2,3,4] and identification operations [5,6,7,8]. Underwater manipulators comprise multiple groups of rigid bodies, which are connected through a series of joints. Some underwater manipulators are equipped with specialized equipment such as underwater fill lights and cameras, can perform tasks that include cleaning and opening cables [9,10], inspecting underwater surfaces, and performing other manual tasks [11,12,13,14]. Similarly, there are many motion controls for underwater manipulators [15,16,17,18,19,20]. However, an underwater manipulator will generate a reaction force, which will cause an impact on the stability of the underwater robot. In this way, a stability compensation controller is added to the control system of the underwater robot that enhances the complexity of the control system of underwater robots, making it challenging to implement an underwater acquisition and identification task. To reduce the complexity of the system and the flexibility of the manipulator, a novel father–son underwater robot system, where the son underwater robot as a novel manipulation system concept, was proposed in this paper. A father–son underwater robot system is a type of multi-robot system that can combine the functions of two different types of robots. Multi-robot systems can combine the advantages of different types of robots into one platform.

The current underwater target acquisition technology mainly uses large-scale industrial platforms suitable for deep-sea industrial technology. However, large industrial platforms are not suitable for use in shallow sea areas. Moreover, large industrial platforms may damage the ecological environment for some particular areas, such as coral areas. The MARIS project has developed technologies and methodologies of the autonomous underwater vehicle manipulator systems. The total weight of the MARIS system ranges from 350 to 500 kg. The control mechanism can be operated with six degrees of freedom [21,22]. The TRIDENT project was a project that aimed at developing autonomous intervention tools that can autonomously search and recover items. The TRIDENT I-AUV is a lightweight system that can be used for projects with a weight of less than 200 kg [23]. Most lightweight ROVs are equipped with low-degree-free manipulators, which are not ideal for operational flexibility. A two-degrees-of freedom (DoF) joint underwater mechanical manipulator was proposed, its prototype can generate motion in the air and underwater using piezoelectric materials [24]. In [25], a novel underwater robotic system that uses the soft robots’ unique characteristics to improve its rigidity and force output was proposed. It features a hybrid structure that combines a soft frame and a rigid hydraulic actuation system. A cable-driven underwater manipulator based on the buoyancy regulation system controller controls the buoyancy of the various sections by varying the static and dynamic states of each section [26]. Based on our analysis of the latest research, from the perspective of technical contributions, our developed father–son underwater robot system (FURS) shows good motion performance and flexibility in shallow water. It also has the function of acquisition and identification of target object image information. The proposed FURS shows the potential for the implementation of underwater tasks.

**Figure 1:**
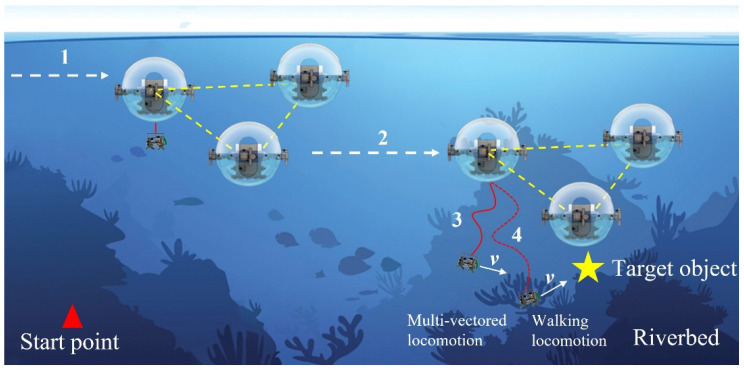
Schematic of the operation principle of the father–son underwater robot.

In our previous research, we first proposed the Father–Son Underwater Robotic System (FURS) [27]. The amphibious underwater robot is the father underwater robot of the FURS that provided the motion performance on land and underwater. An ICPF actuators-based micro-robot plays the role of the son underwater robot. The amphibious underwater robot developed in our laboratory has developed motion control and realizes the underwater communication between each robot [28,29]. At the same time, we also implement many different functions, such as path planning, the function of the localization with the method of relative close-range, and trajectory tracking. There is also a spherical underwater robot that has good flexibility and good hydrodynamic performance in our laboratory. SUR III [30] and SUR IV are equipped with four thrusters at the junction of the two hemispheres and realize an underwater speed switch of high speed and low speed after SUR II. SUR V [31] realizes the collaboration control between multiple robots while realizing speed switching. Moreover, it can achieve long-distance and long-term movement. Based on the characteristics of SUR V, the SUR V has been chosen as the father underwater robot for the FURS. The novel FURS was proposed in this paper to achieve good motion performance and underwater target object acquisition and identification ability. In the collaborative task carried out by the multi- SUR Vs, the son underwater robot is equipped on the SUR V as a manipulator to complete the acquisition and identification task of the underwater target object. Taking one of the spherical underwater robots equipped with a son underwater robot as an example. The spherical underwater robot has the characteristics of long-range and fast movement. When the multi-SUR Vs moves near the target area, the robots first cruise and surround the target object. Then, the robot carrying the son underwater robot opens the coiling mechanism and releases the son underwater robot. The son underwater robot moves close to the target object and identifies the target object through the camera and acquisition image information about the target object. The schematic of the operation principle of the father–son underwater robot as shown in Figure 1.

With the aim of underwater target objects acquisition and identification in shallow waters, a novel bio-inspired father–son underwater robot system is developed in this paper. Compared with other conventional manipulators [32,33,34], our proposed FURS uses high-performance hybrid thrusters that consist of the two propeller thrusters and water-jet thrusters of the father underwater robot, which was proposed in previous studies, showing better swimming abilities in underwater environments. With the help of hybrid thrusters, the FURS can approach the underwater target object quickly using propeller thrusters, and then, the multi-robot cruises around the target using the water-jet thruster due to its relatively low-speed undulation. Meanwhile, water-jet thrusters can contribute to the son underwater robot by underwater manipulation. A Salamandra-inspired son underwater robot is developed as the manipulator of the FURS, which is connected to the spherical underwater robot by the tether. The son underwater robot has multiple degrees of freedom and has realized both swimming and walking movement modes. That means the son underwater robot can reach difficult-to-reach areas underwater, such as coral areas. Moreover, a coiling mechanism was designed for the recycling and release of the son underwater robot. An underwater vision system with a monocular camera is proposed to realize the identification of underwater target objects. Overall, the relevant experiment verifies the capacities for underwater target object acquisition and identification of our FURS proposed in this paper of the proposed technology.

The remainder of this article is organized as follows. Section 2 introduces the design and control mechanism of the father–son underwater robot system. Analysis of the father–son underwater robot system introduces in Section 3. Underwater characteristics evaluation experiments of the father–son underwater robot system introduce in Section 4. Finally, Section 5 concludes this paper.

## 2. Design and Control Mechanism of the Father–Son Underwater Robot System

This section presents the mechatronics of the FURS. A detailed description of the SUR that the father underwater robot of the FURS, and the bio-inspired son underwater robot as the manipulator subsystem of the FURS, is given since all the work was developed by us. The control architecture designed for our FURS is also presented in this section. The control consists of four parts: the SURs control, the son underwater robot, the coiling mechanism, and the vision system.

### 2.1. Overview of the Father–Son Underwater Robot System

**Figure 2:**
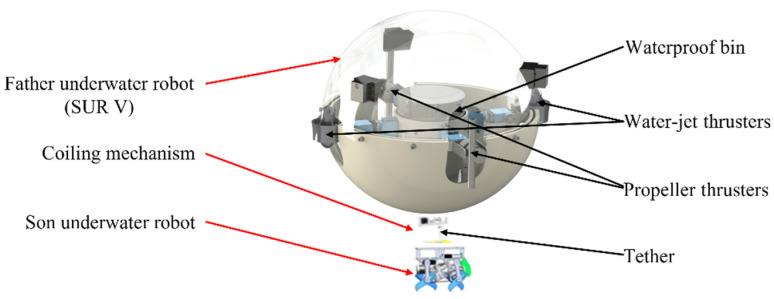
Three-dimensional model of the FURS system.

Symbiosis, a close and mutually beneficial relationship between two different organisms, is often found in nature. Symbiosis exists between animals, plants, fungi, and any two of the three. In a symbiotic relationship, one species provides survival assistance to the other and receives help. The two species live together and depend on each other for their mutual benefit. Although the robots face different challenges, the same principles can be applied in which the strengths of each of the two robots can compensate for each other’s weaknesses. Our FURS combines the strengths of both robots and is designed explicitly for target object acquisition and identification in underwater difficult-to-reach locations. There are three parts of the FURS that the father underwater robot, the bio-inspired son underwater robot, and the coiling mechanism have consisted of the total system, as shown in Figure 2. The FURS combines the advantages of the father underwater robot and son underwater robot. The father underwater robot of the FURS that can move to specific areas and SUR V proposed in our Lab has been chosen for the father underwater robot. SUR V has a strong dynamic balance to resistance to underwater environments features undercurrents. Moreover, the SUR should have good maneuverability to compensate for the effects of undercurrents when the son underwater robot acquires the target object. The SUR V is a spherical underwater robot with a self-protect hull. Its design was proposed to protect the inner structure of the robot from external damage, and the spherical structure can realize a zero turning radius smoothly. The bio-inspired son underwater robot of the FURS, which acquires and identifies the target object. Considering the specific situation of the son underwater robot, it has a compact structure and a variety of motion modes, which can approach the target object stability. We will introduce the son underwater robot of FURS in detail in Section 2.2. The coiling system can recycle and release the son underwater robot. The tether consists of a 2.3 mm outer diameter silicone tube that shields the cable from seawater and is bonded at its extremities. It contains a cable of tinned copper and is chosen with a 22 A specification. The depth constraint of the son underwater robot depends on the length of the tether. To have an operation envelope of a spherical cap of 3 m radius for the FURS, an equivalent length of tether needs to be reliably coiled before the movement. The end of the tube is sealed onto the upper resting in the son underwater robot.

Natural sea environments typically feature undercurrents, especially on the shallow waters. Underwater vehicles should have good maneuverability to compensate for the effects of undercurrents. The FURS, which combines the advantages of the father underwater robot and the son underwater robot, which can approach the underwater target object quickly and target object acquisition and identification in underwater difficult-to-reach locations. The FURS is actuated by the symmetrical water-jet thrusters and propeller thrusters to adapt to the complicated sea environment by using the propeller thruster to generate large propulsive forces for quickly cruising and approaching target objects. Compared with propeller thrusters, these achieve more precise position control due to producing smaller propulsive forces. This means the water-jet thrusters can realize the fine pose the SUR V requires, enabling the robot to approach the target objects stably. The 6-DOF underwater manipulator can receive the underwater target object and identify its information. Moreover, the 6-DOF underwater manipulator can be recycled and released. The technical specification of the FURS is shown in Table 1.

**Figure 3:**
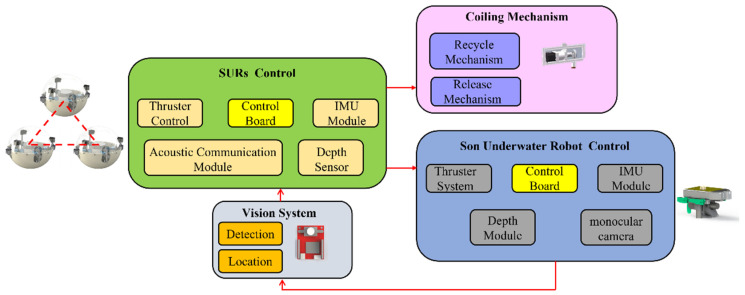
The control architecture of the FURS.

The control mainly consists of four parts: the SURs control, the son underwater robot, the coiling mechanism, and the vision system. The main controller is an AT Mega 2560 (Arduino, Italy), which coordinates with the sensor module to collect data about the robot’s performance. The motion of the FURS can be measured by an inertial measurement unit (IMU) and depth sensor. The underwater vision system consists of a camera that quickly detects and identifies target objects. Multiple SURs rely on acoustic communication modules for information transfer. All control circuits are collected in the waterproof bin. Moreover, a coiling mechanism was equipped that realizes the recycling and release of the 6-DOF underwater manipulator. As described above, the objective of the FURS is the acquisition and identification of target objects in underwater difficult-to-reach locations. The hybrid thruster system can propel FURS closer to the region of the target. The proposed son underwater robot can approach the target objects using the motion control of walking and swimming. The coiling mechanism control can realize the recycling and release of the son underwater robot. The vision system can facilitate the acquisition of the image information as well as the identification of underwater target objects. The control architecture developed for the FURS is shown in Figure 3.

### 2.2. Vision System of the Father–Son Underwater Robot

**Figure 4:**
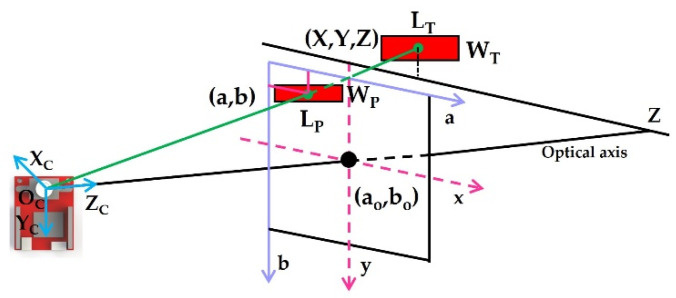
Transform the coordinate system.

The vision system of the FURS was equipped with an Open MV4 camera (monocular vision). The height angle and horizontal angle of the camera view were 115° and 90°, respectively. A customized waterproof hull was made using 3D printing technology and mounted in front of the robot head by the bracket. Optical glass was fixed in the front of the camera lens that reduced the impact of the waterproof hull on image clarity. The Open MV4 camera can quickly detect and identify target objects due to its large field of view. We could also obtain the approximate positions of target objects through the monocular camera if target objects were detected. Various studies have been conducted in order to develop a better and more accurate monocular ranging system. In this paper, the distance between the target object and the reference can be calculated using the monocular passive ranging method. The 3D coordinates of the target object can be determined by,

(1) $$\{\begin{array}{c} Z=\left(\frac{L_{T}f}{L_{p}}+W_{T}f/W_{P}\right)/2\\ X=Z\left(a-a_{O}\right)dx\\ Y=Z\left(b-b_{O}\right)dy \end{array}$$

where *f* is the focal length of the camera. *X*, *Y,* and *Z* are the *X*c-, *Y*c-, and *Z*_c_-direction component values in the camera coordinate system, respectively. *L_T_* and *W_T_* are the length and width of the target object, respectively. On the pixel plane, the length and width of the target object are *L_P_* and *W_P_*. (*a_O_*, *b_O_*) are the coordinates of the origin of the image coordinate system in the pixel coordinate system. *dx* and *dy* are the physical dimensions of each pixel in the a- and b-directions of the image plane, respectively. Finally, we can obtain the 3D coordinates of the target object in the camera coordinate system. The target object transforms the relationship in the coordinate system, as shown in Figure 4.

### 2.3. Design of the Bio-Inspired Son Underwater Robot

**Figure 5:**
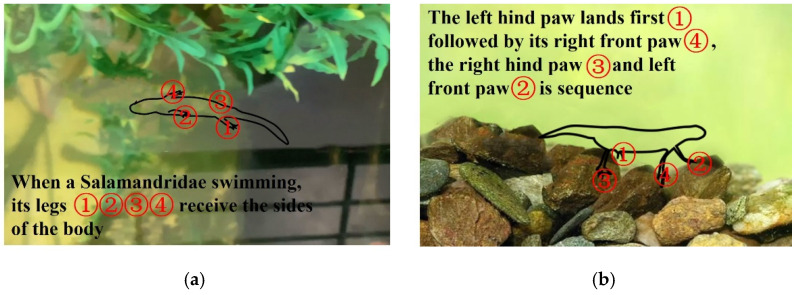
Illustration of Salamandra motion: (**a**) illustration of Salamandra swimming; (**b**) illustration of Salamandra swimming.

**Figure 6:**
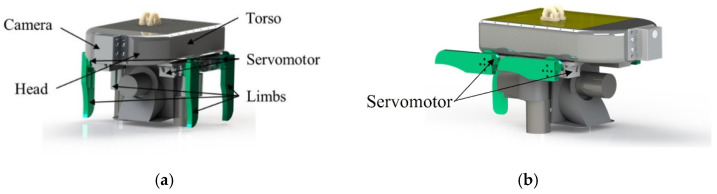
Mechanical design of the bio-inspired son underwater robot: (**a**) design of the son underwater robot; (**b**) illustration of the son underwater robot swimming.

Salamandra inspired the son underwater robot of the FURS, which has multiple locomotion modes. It depends on its legs to walk underwater, as shown in Figure 5b, and it will retract its legs when it swims underwater, relying on the swing of the tail and the strength of the body to move, as shown in Figure 5a. The illustration of Salamandra motion mode is shown in Figure 5. The body length of the son underwater robot is about 180 cm, the body width is about 120 cm, and the body height is about 30 cm. The height of the limb is 51 cm. The son underwater robot’s torso, limbs, tail, and head are 3D printed with PLA material. Each of the limbs is controlled by a servomotor. Battery and sensors are all installed inside the torso, protecting them away from water. The two-servo motor for controlling the tail is installed on the backside of the torso to realize the control of the movement direction of the son underwater robot. A camera (monocular vision) is installed at the front of the head of the son underwater robot for the acquisition and identification of the underwater target object. The underwater vision system consists of an Open MV camera that provides a large underwater environmental field of vision for the son underwater robot to acquire and identify the underwater target object. The design of the son underwater robot is shown in Figure 6. When walking on the riverbed, the rotation angle of the leg ranges from 0° to 45°; we set the installation angle of the leg to be 0°. The gravity line passes through the limb’s center point and ensures the installation’s stability for the son underwater robot. The length of the tail is about 30 cm, which is one-sixth of the body’s length. The tail can be used as a rudder to control the swimming direction. A waterproof servomotor actuates the robot’s tail with a rotational range between 0° and 180°. As for the swimming motion, limbs rotated to both sides through the servomotor, as shown in Figure 6b. When the robot switches swimming mode, water flows into the body. Through the water inlet so that the tail cavity of the robot is filled with water. The forward and reverse DC reducer drives the blades and rotates so that the front part and the whole accessory structure can be forced to move forward. The swinging blades mainly control the movement direction of the robot. Two DC pushrod motors can alternately drive the swinging blades forward so that the forward direction of the robot can be controlled. In the vertical direction, similar to the forward movement, two propellers are used to achieve the ascent and descent movement of the robot. The technical specification of the son underwater robot is shown in Table 2.

**Figure 7:**
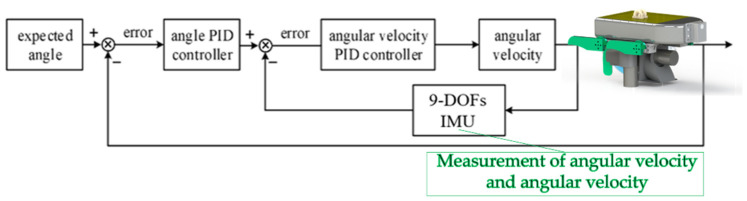
Transform relationship of different coordinate systems.

The data of attitude of the son underwater robot can be collected by a 9 DOFs IMU (WIT, China) and a depth sensor. The son underwater robot is equipped with an Arduino nano 33 BLE Sense (Arduino, Italy), which receives feedback from the sensor. The input quantity of PID control is based on the error between the feedback quantity and the desired value. The son underwater robot can resist external disturbances controlled by a cascade PID control self-control system. The angle/angular velocity-cascade PID control system enhances the anti-interference of the system. Compared with a single controller, the cascade PID control system can control more variables, making the robot more adaptable. The cascade PID control self-control system of the son underwater robot is shown in Figure 7.

## 3. Analysis of the Father–Son Underwater Robot System

This section presents the analysis of the father–son underwater robot system. The FURS incorporates the characteristics of both robots, and to better show the advantages of both robots, we analyze the characteristics of both robots separately. The kinematic and hydrodynamic analyses of the son underwater robot show its dynamic stability during walking and swimming. The stability analysis of the father underwater robot shows the anti-interference ability of its motion.

### 3.1. Kinematic Analysis of the Bio-Inspired Son Robot

In the analysis of swimming postures of underwater animals, the main categories are as follows: “paddling style” such as fish swimming by the swing of the carapace and fins; “bouncing style” such as the arthropod shrimp, swimming by the paddling of the lamellar swimming foot; “breaststroke” such as adult frogs, the front legs in front of the symmetrical chest side down flexion paddling, the hind legs symmetrical flexion and extension stirrup clip water, to obtain forward propulsion, and so on. Moreover, many creatures walk on the seafloor. The son underwater robot was proposed in this paper was inspired by salamanders. Salamanders are amphibians; they can swim and walk on land and freely switch between these two modes of movement. The multi-motions mode increases the flexibility of movement. We have analyzed the son underwater robot’s walking and swimming motion mode and summarized their locomotor posture by modeling.

**Figure 8:**
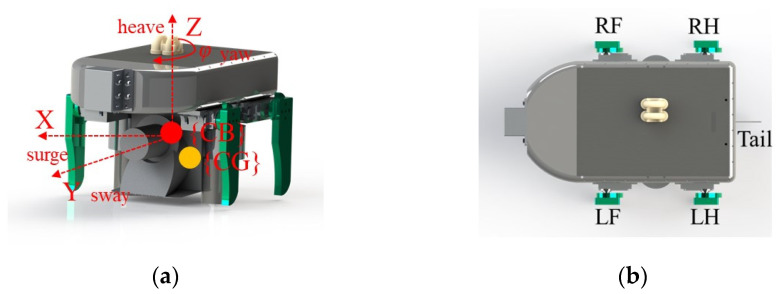
The bio-inspired son underwater robot: (**a**) relative positions of CB and CG; (**b**) exterior of the proposed son underwater robot. The right foreleg, right hind leg, left foreleg, and left hind leg are indicated by RF, RH, LF, and LH, respectively.

**Figure 9:**
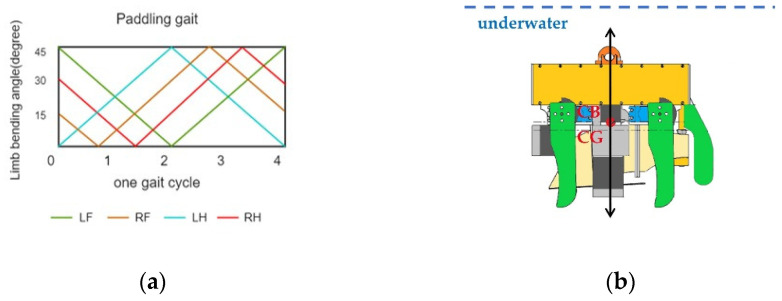
Analysis of a walking cycle: (**a**) limb motion in a walking cycle; (**b**) floating body stability.

The degree of freedom reflects the degree of flexibility of the underwater robot. The design of the degrees of freedom directly affects the dynamic stability of the son underwater robot in the water. Four degrees of freedom (DOFs) are designed based on the spatial coordinate axes: surge, sway, heave, and yaw, as shown in Figure 8a. The analysis of the kinematic of the salamander shows that the movement of the salamander’s limbs while walking in the water is sequential so that the body’s dynamic balance in the water can be maintained. When the limbs are walking in the water, the walk order is left front (LF)—right hind (RH)—right front (RF)—left hind (LH) as shown in Figure 8b, such an order can make the movement more coordinated, to avoid the same side of the limbs affect each other. When designing the son underwater robot, the mechanical limbs of the son underwater robot are also designed in this order of motion. The activity angles of the limbs designed in this paper are from 0° to 45°. Taking the LH limb as an example, the horizontal coordinate (0, 1) corresponds to the vertical coordinate (0°, 45°), which indicates the meaning of the LH starts to move forward. At this time, the LF limb returned from 45° to 0°, while RF and RH performed the corresponding movements, respectively. The motion state of each limb is shown in Figure 9a.

The ability of the son underwater robot to float in the water depends mainly on the design of the center of gravity (CG) and center of buoyancy (CB). The floating center is the shape center of the submerged part of the volume of the submerged body. Since the robot is completely submerged, the floating center of the robot remains constant, so we define the CB of the son underwater robot as the origin of the coordinate XOY, as shown in Figure 9b. As the position of the limbs will make the center of gravity of the son underwater robot change, the relative position of the buoyancy center and the center of gravity will change. Only the CG and CB of the son underwater robot are in the same position, and the son underwater robot can achieve dynamic equilibrium when working in water. The son underwater robot will keep the balance under combined torque of the force of buoyancy and gravity. Based on the moment of CG to CB during walking, we can estimate the robot’s stability. We define x and y as the relative displacement of CB to CG. For the y-direction, it is less likely to cause a change in pitch angle so that the change in CG is relatively tiny. To verify whether the angular roll shift occurs in the x-direction, causing the robot to be unbalanced, we calculate the moment in the x-direction. Taking the first step of the walk cycle as an example, the moment can be approximately represented as |X*cg*1| × *f_B_*. As a result, the moment is 0.032 nm. The 0.035 nm, 0.042 nm, and 0.031 nm in the other three steps, respectively. Because the length of the son underwater robot is 0.18 m, the influence of the moment is not obvious in walking motion.

### 3.2. Hydrodynamic Analysis of the Bio-Inspired Son Robot

**Figure 10:**
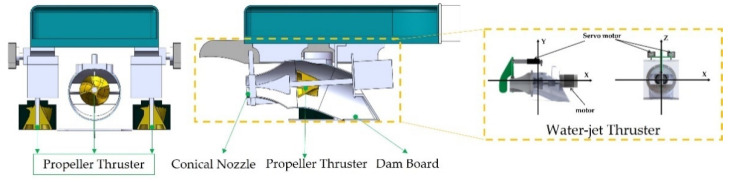
The cutaway view of the son underwater robot.

The hydrodynamic characteristics of a robot are also important factors that determine the efficiency and accuracy of its control algorithms. When the son underwater robot performs walking motion, the motion of the legs of the son underwater robot is controlled by four servos that is the main power structure. The servos can make the son underwater robot perform the linear motion, and the ground support enables the son underwater robot to perform walking motions on the riverbed. In the swimming motion mode, the linear movement of the son underwater robot is caused by the horizontal blade assembly on the son underwater robot spinning the water to move the torso. When swimming in the water, the water flows into the body through the water inlet, making the inner cavity of the tail of the son underwater robot full of water, and the forward and reverse DC reducer drives the blades and makes rotational movement, so that it can drive the front part and the overall attachment structure to move forward. The oscillating blades mainly control the movement direction of the son underwater robot, and the servo motor can drive the turning of the oscillating blades so that the forward direction of the son underwater robot can be controlled. The depth motion is made by the vertical blade assembly on the son underwater robot spinning the water to make the body move. The cutaway view of the son underwater robot is shown in Figure 10.

**Figure 11:**
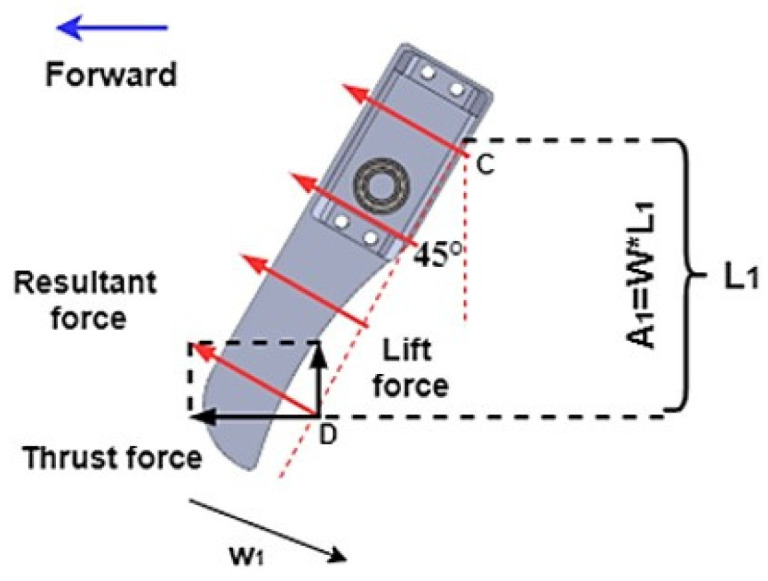
Schematic diagram of the forces during limb walking.

**Figure 12:**
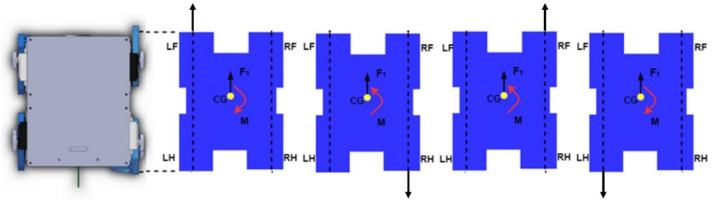
Limb forces in a walking cycle.

First, the hydrodynamic analysis during the walking motion was carried out. The reaction force generated by the water on the limb (red arrow on the limb in the figure) is uniformly distributed along the limb when the limb is moving backward (w1 direction). An upward-lifting force (Lift force) and a forward thrust force (Thrust force) can be obtained by decomposing the reaction force. The upward lift force can be used to supplement the buoyancy force, making it possible to maintain the dynamic balance of forces on the son underwater robot during operation. In order to enable the son underwater robot to move forward, the thrust force of the robot should be higher than the drag force of the water. Figure 11 represents the forces on one of the limbs of the son underwater robot during the walking process in moving forward, which focuses on the analysis of the thrust force. The thrust force is calculated in the water as shown in Equation (2),

(2) $$F=\frac{1}{2}C_{d}\left(R_{e}\right)\rho V^{2}A$$

where *F* is the resistance or thrust in the fluid (here, the fluid is water), *R_e_* is the Reynolds number, *ρ* is the density of water, *V* is the relative velocity of the robot to the fluid, *C_d_* is the drag coefficient, and *A* is the reference area (the positive projection of the limb in the direction of motion). Moreover, the drag coefficient is determined by the Reynolds number *R_e_*,

(3) $$R_{e}=\frac{\rho VD}{\mu }$$

(4) $$C_{d}=\frac{2T}{\rho V^{2}A}$$

where μ is the viscosity coefficient, which is $1\times 10^{-6}$ at 200 °C. The maximum simulation velocity of the robot is 0.15 m/s in horizontal directions. By calculating in (3), the Reynolds number is *R_e_* = 0.6 × 10^5^, which indicates that the flow is turbulent when the robot moves through the water. The drag coefficient is *C_d_ =* 0.82. When the limb moves forward, the projected area is *A = W × L (*α*)*. The rotational speed of the servo motor is *n =* 0.20 s/60°, and the angular velocity of the limb can be obtained by converting the rotational speed of the rudder and the angular velocity of the limb. For a given position on the limb, the flow velocity can be approximated by *u = Rω*, where *R* denotes the distance from the point on the limb to the winding point. The walk cycle is divided according to the different positions of the robot’s limbs. As shown in Figure 9, one limb provides thrust in every 1/4 cycle. The thrust of the son underwater robot is provided by LH, FF, FH, and LF in turn. In the process of calculating the thrust force of the limb in the water, the force at different locations will vary due to the different water flow velocities at different locations. The force on the son underwater robot’s limbs during the walk cycle is shown in Figure 12.

We assume that a certain point, the formula for calculating the thrust and drag force at a certain point is as follows

(5) $$F_{thrust}=\int_{0}^{45}\frac{1}{2}C_{d}\left(R_{e}\right)\rho V^{2}A=\int_{0}^{45}\frac{1}{2}C_{d}\left(R_{e}\right)\rho {\left(u\left(\alpha ,l\right)-v\right)}^{2}W\times L\left(\alpha \right)$$

where α is the rotation angle, *l* is the distance around the rotation point, *v* is the velocity of the son underwater robot walking velocity at the time of paddling, u(α,l) is the relative horizontal thrust velocity of the point per unit length limb at rotation angle *α*, and L(α) is the vertical projection length of the powered limb at α. According to the geometric relationship,

(6) u(α,l)=ωl(α)

(7) $$L\left(\alpha \right)=CD\frac{\alpha }{45}\cos\left(\alpha \right)$$

(8) ω=2πn

where CD is the length of the limb, n is the rotational speed of the servomotor. Based on the above equations, Equation (8) is substitution into Equation (6), and then Equations (6)–(8) are substitution into Equation (5). The expressions for the thrust force can be deduced as follows

(9) $$F_{thrust}\left(\alpha ,l\right)=\int_{0}^{45}\frac{1}{2}C_{d}\left(R_{e}\right)\rho {\left(2\pi l-v\right)}^{2}W\times CD\frac{\alpha }{45}\cos\left(\alpha \right)$$

In Equation (9), the drag coefficient $C_{d1}\approx 0.103$. As the son underwater robot walks forward in the water, the current exerts a backward force on the robot. Now the robot model is simplified to a cube. Thus, the drag coefficient of the torso is $C_{d2}\approx 0.82$. With a constant velocity, the robot receives equal thrust force and drag force,

(10) $$F_{drag}=F_{thrust}$$

where $F_{drag}$ is the resistance received by the torso of the robot. According to Equation (10), the final velocity obtained by the robot.

(11) $$\frac{1}{2}\rho C_{d2}A_{torso}v^{2}=\frac{1}{2}\rho C_{d1}A_{limb}{\left(u-v\right)}^{2}$$

According to Equation (11), the drag force will be greater than the thrust force when *v >* 0.83 cm/s. Therefore, theoretically, the maximum speed of the robot is 0.83 cm/s. After this, the influence of the flow field from the son underwater robot in swimming mode is considered in the hydrodynamic analysis. The model of the son underwater robot is processed first. Some parts such as screws have been omitted and irregular solids have been changed to a regular shape. A rectangle with a length of 1 m, a width of 1m, and a height of 3 m was chosen. Similarly, a cylinder to the outside of the propeller to represent the domain of rotation was added. Due to the hydrodynamic simulation involving propeller rotational motion, the steady-state simulation uses the MRF method. In this simulation, the mesh was divided into the tetrahedral mesh; the total number of elements was 4,848,756, the total number of nodes was 910,456, and the mesh quality was 0.087. To obtain the effect of velocity and pressure of the robot in the flow field, the robot was assumed as a static status, and the flow field environment was 20 °C without external disturbances. The flows field was set with a constant velocity and the velocities of forwarding movement and up movement were 0.15 m/s and 0.1 m/s, respectively.

**Figure 13:**
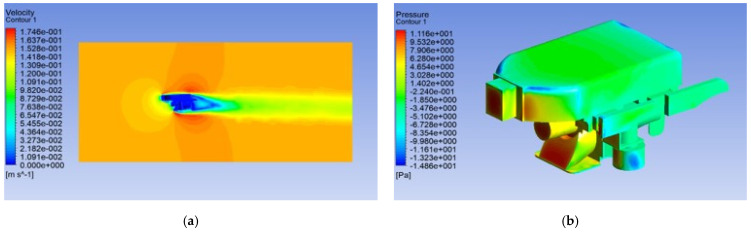
Simulation results in static flow field of forward motion: (**a**) result of the velocity; (**b**) result of the static pressure.

**Figure 14:**
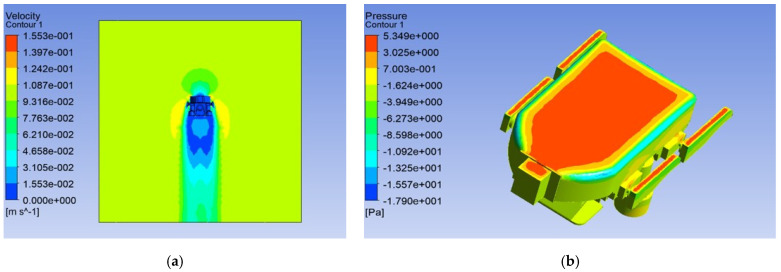
Simulation results in static flow field of floating motion: (**a**) result of the velocity; (**b**) result of the static pressure.

After hydrodynamic analysis simulations, the effect of the impact on the robot structure was not obvious can be ignored. The velocity of the robot was affected by the flow field, as shown in Figure 13a. The maximum pressure near the edge of the robot thruster, a cutaway view of the pressure affected by the flow field, is shown in Figure 13b. Figure 13 shows the simulation result of the forward motion, in here, is the high-speed mode. As for the up movement of the underwater robot, the maximum pressure occurs at the upper part of the robot, and the influence of pressure needs to be considered in the later robot control strategy. The velocity of the robot in the floating motion was affected little by the flow field. The velocity vectors and contours of static pressure for the robot implementing up movement are shown in Figure 14a,b. In the ANSYS FLUENT software, after operating for 1000 steps, the drag coefficients for forwarding movement and counterclockwise movement were constant, converging to *C_d_ =* 0.198 and *C_d_ =* 0.219, respectively; the results of the ANSYS FLUENT analysis were acceptable. According to these results, we can optimize the control strategy of the robot to improve the control accuracy and stability of the robot.

### 3.3. Stability Analysis of the Father Underwater Robot

**Figure 15:**
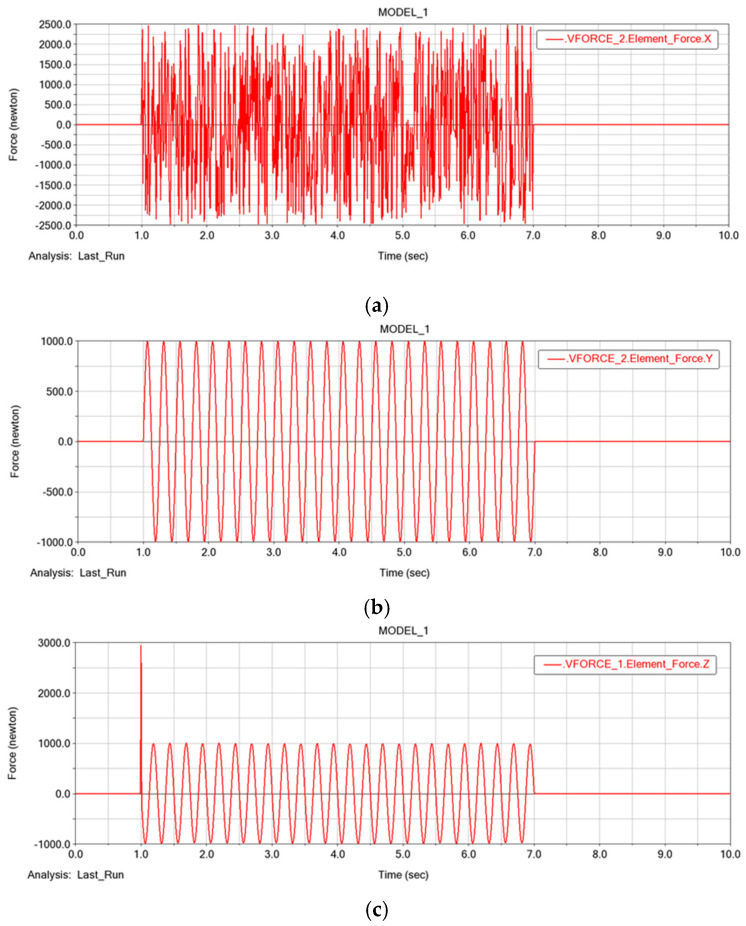
The disturbance force in X, Y, and Z axis: (**a**) the disturbance force in X axis; (**b**) the disturbance force in Y axis; (**c**) the disturbance force in Z axis.

In order to verify the motion performance of the robot, we analyzed the analysis of the father underwater robot in FURS in our previous study [31]. To improve the stability performance of the SUR V, a proportional-integral-derivative (PID) controller was introduced to optimize the robot. The stability controller can adjust the robot’s attitude in time for responding to feedback to further improve the robot’s control accuracy and stability. To verify the stability of the robot’s movement, we modeled a virtual prototype of the SUR under the Adams environment. There are two ways to create a virtual prototype model in Adams: build it in Adams directly or import it using other 3D-modeling software. Due to the complexity of the model and the presence of non-linearities in the simulations, we used the second method. The established model was imported into Adams, and the parts without relative motion in the 3D model were merged and the material properties in the model was set. In this paper, it is assumed that the entire mass of the mechanism is concentrated in the center of mass of the sphere. Since the SUR uses the water-jet thruster and propeller thruster for motion, two different thrusts were added to the robot. Since the underwater environment is complex and there is a large amount of current disturbance. In order to simulate the disturbance force in the underwater environment, random disturbance force was generated using Excel and imported into Adams, which was set as the disturbance force in X axis. Then, the sinusoidal disturbance force set as the disturbance force in Y and Z axis was added. The disturbance force in X, Y, and Z axis is shown in the Figure 15a–c.

**Figure 16:**
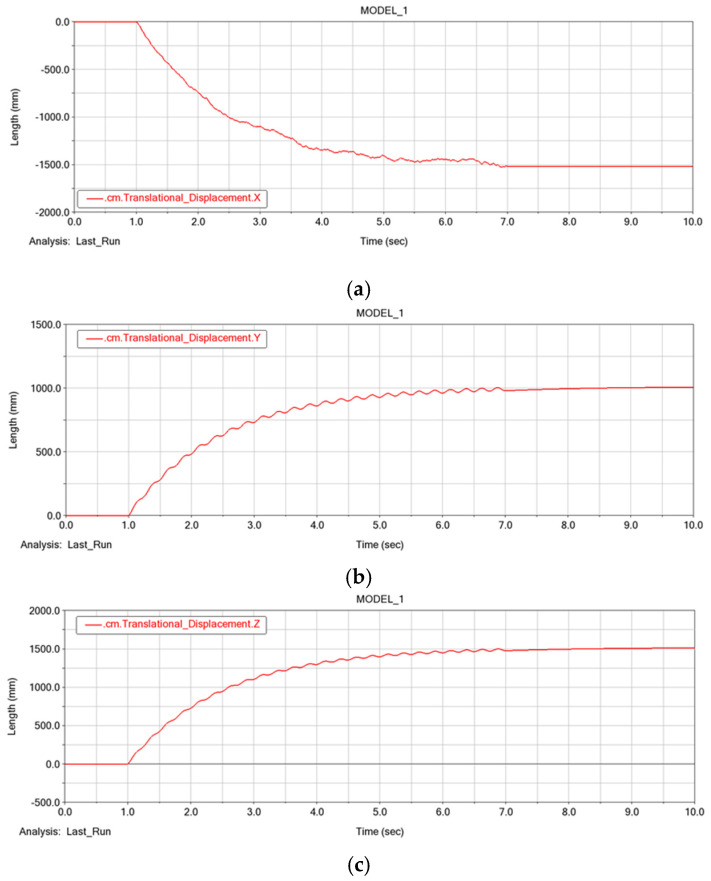
The moving trajectory of the centroid of the robot: (**a**) displacement curve of the centroid in the X direction; (**b**) displacement curve of the centroid in the Y direction; (**c**) displacement curve of the centroid in the Z direction.

Through the post-processing function of ADAMS/View, the moving trajectory of the centroid of the robot was obtained. These movement trajectories also demonstrate the stability of the SUR. We see that the change in the centroid displacement in the X, Y, and Z direction is stable. Although there were some deviations during the movement, the final trajectory still basically followed the initial settings. Consequently, jumping, and sudden changes do not appear, and the robot can implement stable motion. The moving trajectory of the centroid of the robot in X, Y, and Z axis is shown in the Figure 16a–c.

## 4. Experimental Results and Analysis

To verify the effectiveness of the proposed FURS, we conducted a series of experiments in an indoor water tank and an outdoor pool. The experiment consists of the underwater experimental motion control of the son underwater robot and underwater target object acquisition and identification.

### 4.1. Underwater Experimental Motion Control Results of the Son Underwater Robot

**Figure 17:**
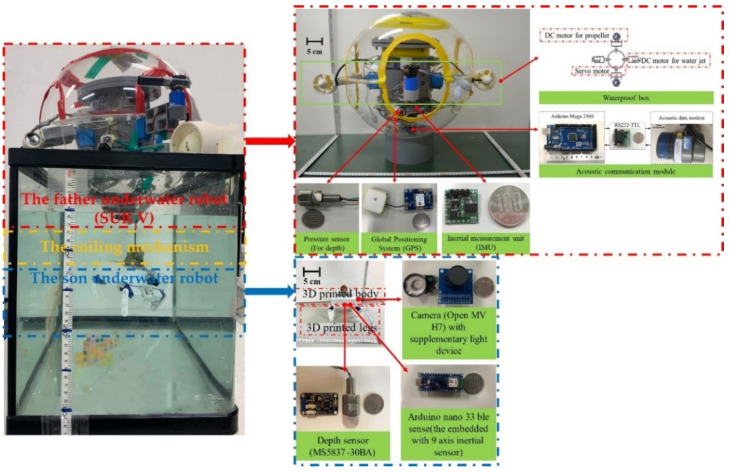
Photograph of the FURS system.

The indoor tests were performed in a 30 cm deep water tank in order to evaluate the performance of the motion of the son underwater robot. The length and width of the water tank were 50 cm and 30 cm, respectively. These swimming experiments involved the forward motion, dive, float motion, and turning movement in the yaw of the son underwater robot, while the son underwater robot always remained attached to the SUR via the tether. Each case was performed five times so as to better verify and evaluate the motion control of the son underwater robot. The resulting curve is shown as the average of the experimental results. The photograph of the father–son underwater robot system is shown in Figure 17, the tether has been partially uncoiled and the son underwater robot is underwater.

#### 4.1.1. Walking Experiments in a Water Tank

**Figure 18:**
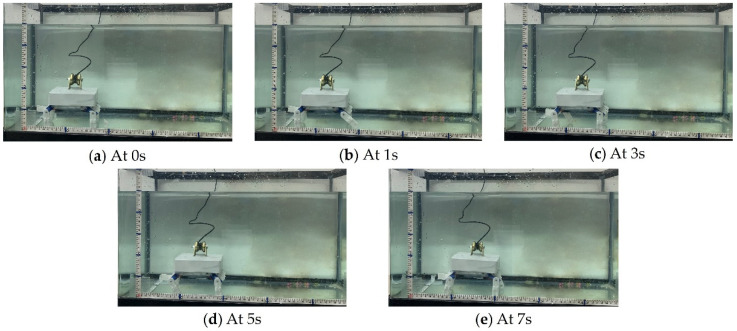
Experiments on walking motion in one walk cycle.

**Figure 19:**
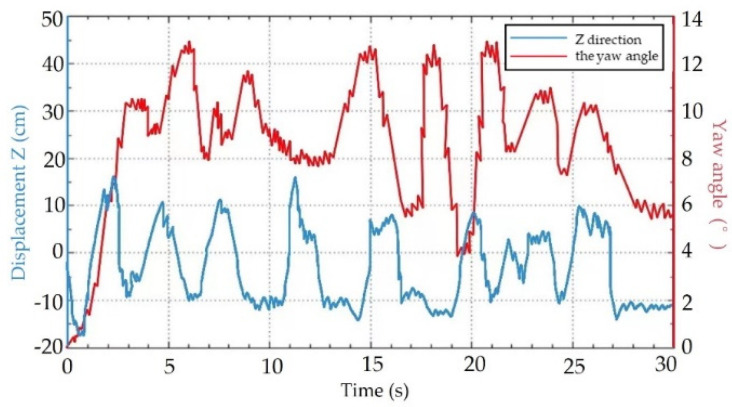
Displacement and yaw angle in the Z direction.

The rhythmic movement of an animal is a manifestation of a high degree of stability and flexibility that can perform various tasks such as walking, trotting, and pacing. Walking motion can realize the possibility of the son underwater robot reaching difficult-to-reach areas where walking is required. Therefore, it is necessary to perform the walking movement of the son underwater robot. Five experiments were carried out in each cycle of the walking motion experiment of the son underwater robot. The experimental data of deviation were collected from IMU and depth sensors. The time and displacement in the walking experiment were recorded, including the displacement in the forward X direction and deviations in the Z direction and yaw angle, which show the changes in displacement in the forward direction (X) over time. The average walking speed of the robot was about 0.57 cm/s. The maximum deviation in the robot for the Z direction was 8.56 cm, and the maximum deviation in the experimental results for the yaw angle was less than 13.1°. The servo motor was controlled by adjusting the duty ratio of the PWM control signal, and that of the deviation between the actual rotation angle of each steering gear and the theoretical value rotation angle. In the real experiment, there is some deviation in the Z direction and yaw angle. Since there will be a gap between the son underwater robot’s feet and the ground, slipping may occur, which causes the walking speed to be lower than the simulated value. This is another reason for the deviation in the Z direction and yaw angle. There also may be delays in the control signal, which may also cause deviations. However, the overall walking deviation of the robot is low, which achieves stable crawling. The progress of the walking movement of one walk cycle for the son underwater robot is shown in Figure 18. Figure 19 shows the experimental results of walking motion.

#### 4.1.2. Swimming Experiments in a Water Tank

**Figure 20:**
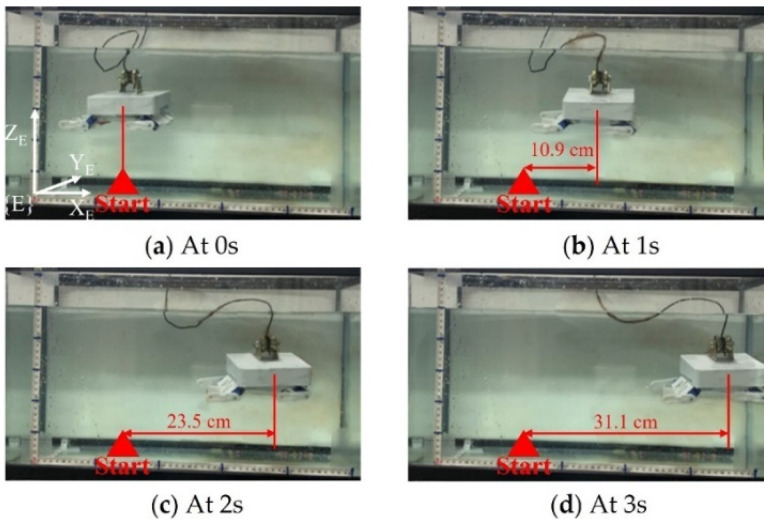
Experiments on forwarding motion in a swimming motion.

While large horizontal distances are covered by displacing the SUR itself, it is essential that the son underwater robot can move within the target region that the SUR is difficult-in-reach. The horizontal movement ability of the son underwater robot can be used to execute underwater tasks in a particular target area. Such as, they can be used to facilitate the acquisition and identification of the underwater target object of the coral reef area. In this paper, the performance of the forward motion of FURS was tested. During the swimming exercise, the robot retracted its limbs to the sides of the body. The son underwater robot moved in a forward motion in the water tank by using the water-jet thruster. The experiment of the son underwater robot forward motion was taken five times. The average velocity of the son underwater robot was calculated as 10 cm/s. The yaw angle changed less than 5°. The forward speed was stable during the whole forward process, and there was no obvious sudden change in rate. The experiment verifies that the water-jet thruster has good motion performance, and the robot can achieve stable forward motion. The experimental results as shown in Figure 20.

**Figure 21:**
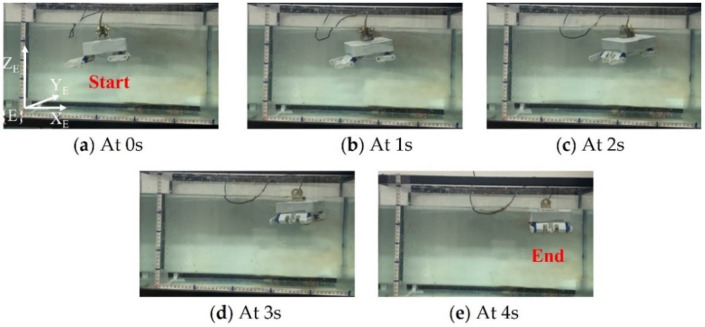
Experiments on turning left motion in a swimming motion.

For the son underwater robot, the turning motion is also one of the required underwater motion modes. The robot can achieve turning movement by adjusting the swing direction of the tail through the servo motor, and the water-jet thruster provides the motion forward. Each experiment of the son underwater robot turning motion was taken five times. In the left turning experiments, the robot completed a 90-degree turning in 4 s with an average speed of 22.5 °/s. As for the right turning experiments, the average speed of 18 °/s is in total progress. The reason for this discrepancy may be a delayed signal from the servo when making a right turn. The average value of the rotation radius of the robot during the turning motion was 21.3 cm. Figure 21 records the process in which the SUR IV turns the left movement of 90°.

**Figure 22:**
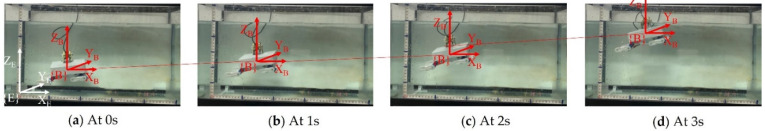
Experiments on floating motion in a swimming motion.

**Figure 23:**
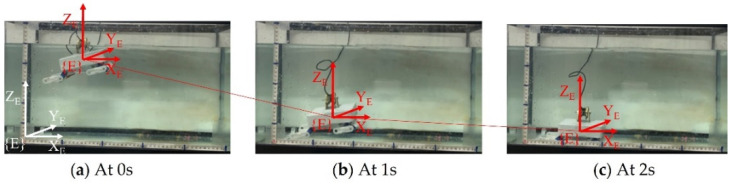
Experiments on diving motion in a swimming motion.

For the dive and float motion, two propeller thrusters will work together. To make the son underwater dive and float underwater stability, the relationship between buoyancy and gravity was considered at the beginning of the design, as introduced in Section 2. At the same time, they ensured that the robot floated underwater; the CG and the BG were at the same point. The center of the robot body was set as the reference point for recording the experimental data. The son underwater robot dive and float motion experiment was taken five times. The son underwater robot could achieve stable dive and float motion. The average speed of the float motion was almost 3.3 cm/s, and the average speed of the dive motion was nearly 7.5 cm/s. At the same time, the defection of the robot in pitch and roll was small enough to be negligible in heave motion. The experimental results are illustrated in Figure 22 and Figure 23, respectively.

### 4.2. Underwater Target Object Acquisition and Identification Results

The outdoor tests were performed in a swimming pool with the 3.0 m × 2.0 m × 0.6 m (the length × the width × the depth). The performance of the accuracy motion and carrying capacity of the father underwater robot was evaluated in the previous research [31]. The performance evaluated experiments that involved the anti-interference capabilities, forward motion, and the rotation motion in place. In the high-speed motion experiment, the motion of the FURS by using the propeller thruster, which can be adjusted by PID in the presence of interference to maintain a high-speed straight forward and quickly reach the target area. The total movement distance of FURS forward motion is 3 m and the movement time is 18 s. As for the rotation motion in place experiment, switch to the water-jet thruster, which can be rotated to a specified angle. The FURS can reach the position of 90° within 2 s and remain stationary.

**Figure 24:**
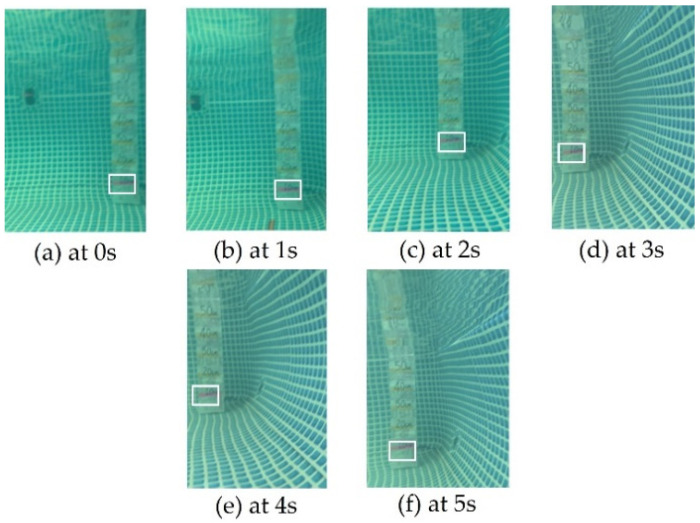
Experimental results for the son underwater robot target object acquisition and identification. (The white box is the target object).

**Figure 25:**
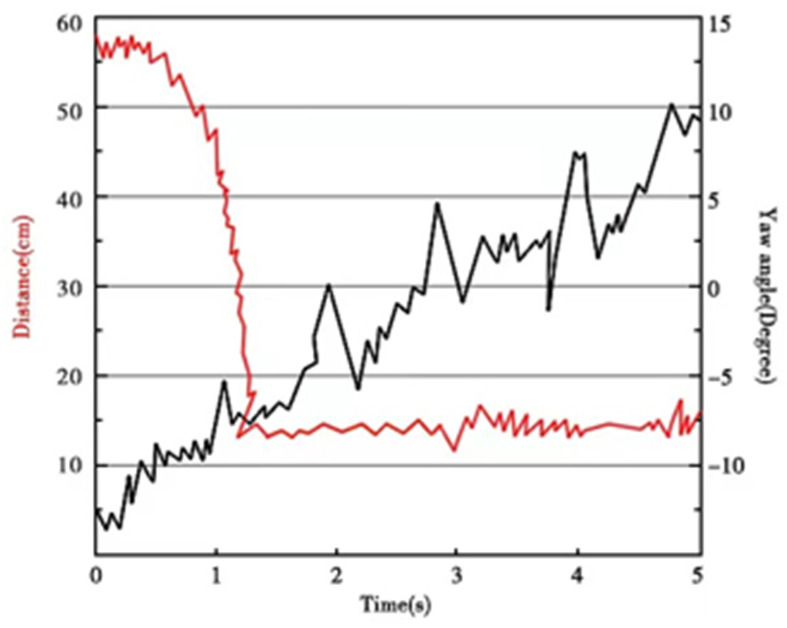
Yaw angle and the dive distance of the son underwater robot.

To verify the effectiveness of the proposed FURS for underwater target object acquisition and identification, we also experimented in the outdoor swimming pool. The experiment of the underwater target object acquisition and identification was taken five times. The formation control method of multi-robots selects the leader–follower control method. The shape of the formation selected in this experiment was a triangle, and the center point of the formation formed by multiple SURs was used as the measurement point. The start point was (0, 0) and the endpoint was (150, 0). The collaboration control experiment was conducted with three robots (Robot 1, Robot 2, and Robot 3). The center point of the formation shape formed by the robots should be from the start point and to the endpoint. Initially, the SUR carrying the son underwater robot opens the coiling structure and releases the son underwater robot. The son underwater robot moves to the vicinity of the underwater target and recognizes the target. After the son underwater robot approached the target object, the acquisition of the target object was obtained by adjusting the yaw angle of the robot. When the target object appears in the field view of the camera, the target is recognized, and the approximate distance to the target is also obtained. The experimental result of acquisition and identification of the target object is shown in Figure 24. From 0 to 5 s, the dive distance of the son underwater robot changed from 58 cm to 15 cm, and the yaw angle changed ±15°. The target object was always in the camera’s field of view during the experiment, and the target object was successfully acquired and identified. The experiment demonstrated the feasibility and reliability of the FURS. The yaw angle and the dive distance of the son underwater robot is shown in Figure 25.

## 5. Conclusions

In this paper, a bio-inspired Father–Son Underwater Robotic System (FURS) is designed for underwater target image information acquisition and identification tasks. The proposed system can rapidly approach the underwater target object using the propeller thruster of SUR that the father underwater robot of the FURS, and then, multi-SURs cruise around the target object by using the water-jet thruster. A Salamandra-inspired son underwater robot for the father–son robot system was developed. The son underwater robot is the manipulator for the FURS. The design of the son underwater robot can approach difficult-to-reach areas in the shallow area, also avoiding the risk of the large manipulator damaging underwater targets. The son underwater robot has four degrees of freedom consisting of the surge, sway, heave, and yaw, and then realizes both swimming and walking movement modes. The son underwater robot has a highly flexible movement performance, demonstrating the feasibility of safely and efficiently reaching the underwater target area. The underwater vision system is proposed for the acquisition image of the target object in real-time. The capacity of the FURS for the acquisition and identification of the target object was successfully demonstrated.

The proposed FURS presents good motion ability and is suitable for the image acquisition and identification of the target object in shallow waters. In future work, we will focus on combining other sensors to achieve more complex underwater tasks, such as grabbing underwater target objects.

## Figures and Tables

**Table 1:** The technical specification of the FURS.

Items	Parameters	Items	Parameters
Dimension (Maximum outer diameter)	54 cm (length) 54 cm (width) 67.2 cm (height)	Sensors	Pressure sensor(MS5837-30BA) 9-DoF IMU Acoustic communication module Open MV camera
Mass in air	9.26 kg	Main processors	AT Mega 2560
Thruster mode	Propeller threster Water-jet thruster		

**Table 2:** The technical specification of the son underwater robot.

Items	Parameters	Items	Parameters
Mass in air	1.26 kg	Processors	Arduino Nano 33 BLE Sense
Body size	180 mm (length) 120 mm (width) 30 mm (height)	Sensors	OPENMV4 H7 Pressure sensor (MS5837-30BA) 9 axis inertial sensor (embedded sensors)
Limb size	51 mm (height)	Max thrust	1.57 N
Tail length	30 mm	Power	3.3 V rechargeable batteries
